# Postural Stability in Parkinson's Disease Patients Is Improved after Stochastic Resonance Therapy

**DOI:** 10.1155/2016/7948721

**Published:** 2016-01-26

**Authors:** Oliver Kaut, Daniel Brenig, Milena Marek, Niels Allert, Ullrich Wüllner

**Affiliations:** ^1^Department of Neurology, University of Bonn, 53105 Bonn, Germany; ^2^Neurological Rehabilitation Center, Godeshoehe, 53117 Bonn, Germany; ^3^German Center for Neurodegenerative Diseases (DZNE), 53175 Bonn, Germany

## Abstract

*Background.* Postural instability in Parkinson's disease (PD) increases the risk of falls and is not improved by pharmacological therapy.* Objective.* We performed a double-blind, randomized sham-controlled study to test the effects of stochastic resonance (whole body vibration) therapy on postural stability in PD.* Methods.* Fifty-six PD participants were allocated to either experimental or sham groups. The experimental group received four series of vibration over eight days, with each series consisting of six stimulus trains of 60-second duration using a randomized whole body vibration. Participants allocated to the control group received a sham treatment.* Results.* Within-group analysis revealed that postural stability in the experimental group improved by 17.5% (*p* = 0.005) comparing experimental and sham groups. The between-group analysis of change after treatment comparing both groups also showed a significant improvement of postural stability (*p* = 0.03). Only in the within-group analysis several items were improved after Bonferroni correction, too, rigor 41.6% (*p* = 0.001), bradykinesia 23.7% (*p* = 0.001), tremor 30.8% (*p* = 0.006), and UPDRS_III_ sum score 23.9% (*p* = 0.000), but did not reach the level of significance in the between-group analysis.* Conclusions.* Stochastic resonance therapy significantly enhanced postural stability even in individuals with increased risk of falling. Thus it offers a potential supplementation to canonical treatments of PD.

## 1. Introduction

Biomechanical devices designed to deliver whole body vibration (WBV) are used increasingly to treat neurological impairment of movement. WBV is performed while participants stand on a vibrating platform. Therapeutic vibration can be generated as either a nonstochastic (sinusoidal, nonrandom) or stochastic (nonsinusoidal, random) vibratory pattern. The latter is referred to as stochastic resonance (SR) and the treatment as stochastic resonance therapy (SRT) [1]. The vibrations associated with WBV are believed to induce muscle contractions by stimulating the muscle spindles and alpha motor neurons, thereby producing effects similar to those induced by other, more conventional, training methods [2]. In particular, the vibrations increase proprioceptive sensory input by affecting the Ia and II afferents of various muscle groups, thereby improving sensory system-mediated postural control [3].

We recently observed that SRT improved bradykinesia in Parkinson's disease (PD) patients and gait and speech in patients with spinocerebellar ataxias (SCA 1, 2, 3, or 6) [4, 5]. Moreover, others recently showed that WBV can reduce the risk of falls among community-dwelling older adults [6].

There is weak evidence that randomized vibration therapy may be superior to nonrandomized vibration; this is because nonrandomized vibration is ineffective in PD [7], whereas randomized vibration improves motor function [1, 4]. Thus, we chose to examine more promising SRT in the current study. However, no study has performed a direct comparison of both types of vibration therapy.

Among the core symptoms of PD, postural instability is probably the most relevant one because reduced mobility and falling increase morbidity, leading to a poor quality of life [8–12]. Postural instability is particularly challenging and difficult to treat as it does not respond well to dopaminergic therapy. Indeed, dopaminergic medication can increase some elements of postural dyscontrol [13]. Even deep brain stimulation fails to improve impaired postural stability in PD [14].

To date, no study has confirmed that WBV improves postural stability in PD. In a previous study [4], we showed that postural instability (scored using the pull-test) improved after SRT; however, the results did not quite reach statistical significance. Also, the pull-test may be biased by rater-associated influences. Thus, the present study was based on the use of a standardized mechanical diagnostic technique (dynamic posturography) to assess postural performance and to test it in a new, independent, and larger cohort of probands.

Hence, this study used clinical scores based on balance and dynamic posturography techniques to examine the effects of SRT in PD, with particular focus on postural instability [15, 16].

## 2. Methods

### 2.1. Design

We performed a double-blind two-group design study. Participants were recruited from January 2012 to July 2014. The protocol of this study was approved by the Institutional Ethics Committee of the University of Bonn and all participants gave written informed consent (Lfd. number 069/11).

Participants were allocated to either the experimental or the sham group using a block randomization with an AAABBB distribution model (A = experimental; B = sham). UW, NA, and OK enrolled participants. DB assigned participants to their groups. All participants were blinded with regard to their assignment to the experimental or sham group and the control panel of the SR-Zeptor device® was covered to ensure that the vibration parameters were not visible. The experimental group was treated with four series of SRT on four different days at days 1, 3, 5, and 8 corresponding to Monday, Wednesday, Friday, and Monday at level 7 (corresponding to a spectrum of frequencies where the majority of frequencies applied are at 7 Hz), amplitude 3 mm, including the additional interference function, called “noise” at level 3, consisting of 6 stimuli of 60-second duration; resting time between stimuli was 60 seconds, too. Participants allocated to the sham group received a treatment with the lowest frequency possible (level 1, amplitude 3 mm, no interference function).

All other parameters remained unchanged to avoid any possible confounding factors. All participants were instructed to stand freely on the two platforms with open eyes, regular footwear and to adopt a semisquat position with knees flexed slightly; the vibration is applied on the feet directly. To perform SRT a second-generation SR-Zeptor with interference function (Human Mobility, Berlin, Germany) was used.

### 2.2. Participants

**Figure 1:**
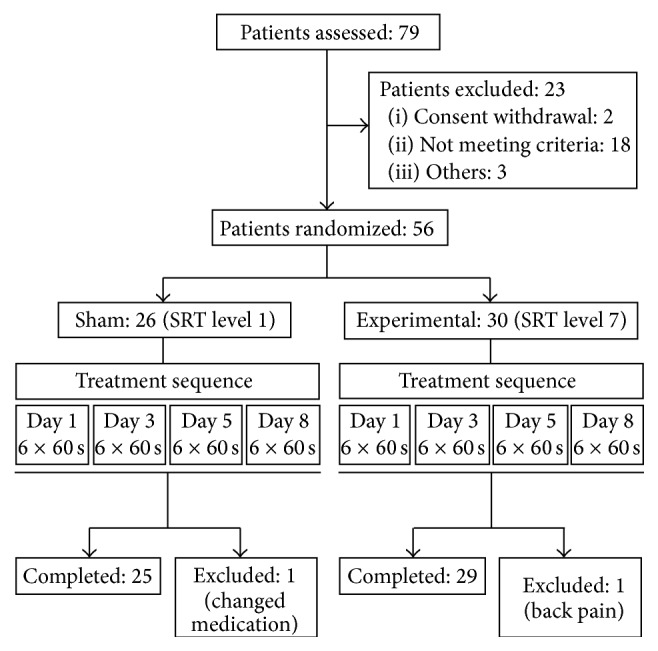
Flow chart of participants included in this study. The numbers indicate the number of patients in each category.

Of 79 patients assessed for eligibility based on their medical records, 56 were finally enrolled and randomized (Figure 1). All 56 participants (male 36; female 20) had idiopathic iPD fulfilling the UK Brain Bank criteria for iPD [17] (either with or without a history of falls) and were recruited from the outpatient clinic of the Movement Disorders Section, Department of Neurology, University of Bonn, Germany, and the outpatient clinic of the rehabilitation clinic Godeshoehe e.V., Germany (details of the study population are summarized in Table 1). Exclusion criteria included atypical or secondary PD, severe dementia, nephrolithiasis, or relevant orthopedic diseases (in particular joint injuries) to ensure greater homogeneity of the population (although none of the above would prevent the application of SRT). Participants were asked not to change their medication during the trial but did not receive any information about the presumed effects of SRT. To reduce any unforeseen risks, we predefined and monitored pain, joint injury, and deterioration of neurological symptoms; patients experiencing these were withdrawn. The study was conducted at the Department of Neurology, University of Bonn, Germany, in accordance with the principles of Good Clinical Practice and with the principles set down in the Declaration of Helsinki.

### 2.3. Clinical Assessments

Functional performance (according to postural stability as assessed by dynamic posturography) was the primary outcome measure [18]. Secondary outcome measures were the pull-test (corresponding to item number 30 of the Unified Parkinson's Disease Rating Scale, part III) [19], timed up-and-go test (TUG; measured in seconds) [20], Tinetti score (a falls risk index used for elderly patients) [21], time needed to walk 8 meters (8MW), and summed scores for UPDRS_III_ items 18 and 19 (speech and facial expression), 20 and 21 (tremor), and 23–26 and 31 (bradykinesia).

Differentiation between PD fallers and PD nonfallers was based on self-reporting: falls were defined as an event that caused the patient to come to rest unintentionally on the ground or lower level [22]. Participants that had fallen more than once during the previous 12 months were allocated to the “fallers” group [8].

Each participant's assessment of their clinical improvement (“better,” “worse,” or “unchanged”) was recorded on Day 8 (after the final intervention). All scores were recorded by a single, blinded, board-certified neurologist who had completed the MDS-UPDRS training program and certificate of exercise (OK). Recordings were made at baseline and after the last treatment (i.e., on Days 1 and 8, resp.). Any adverse events (AEs; muscle soreness, joint pain, or back pain) were also reported by the participants.

### 2.4. Instrument-Based Assessment

Computerized dynamic posturography was performed using an experimentally standardized balance perturbation method, which measured the degree of medial-lateral and anteroposterior sway using an ultrasound-based measuring system with a movable and adjustable plate (PosturoMed CMS10; zebris Medical GmbH, Isny im Allgäu, Germany) [16]. The results of the “provocation test” were analyzed using zebris WinPosture software. Briefly, the movement of the plate was measured by two ultrasound-based sensors attached to the side of the plate. The plate was moved 30 mm to the right relative to the medium position and then fixed in this position by the provocation unit. When the proband stood on the plate, it was suddenly released, thereby generating an unexpected disturbance in stance. The plate then swung back to its resting position. Participants were asked to counterbalance the disturbances using compensatory movements of the legs and body. The movement of the plate was measured until it came to rest, and results were documented as the sum of all sways.

Posturography was performed three times, and the mean of sway was calculated for each individual. Higher values correspond to greater sway, meaning worse clinical performance and impaired postural stability.

### 2.5. Statistical Analysis

Statistical analysis was performed using SPSS 22.0 for Windows (SPSS Inc., Chicago, Illinois, USA). The level of significance was set at *p* < 0.05. Descriptive results are expressed as the mean ± SD. A two-sided paired *t*-test was used to calculate differences between baseline and posttreatment data (UPDRS_III_, TUG, Tinetti score, 8MW, and posturography) for within-group analysis, whereas a two-tailed Mann-Whitney *U* test was used for between-group analysis. Bonferroni correction was used to compare subitems within the UPDRS_III_ (adjusted *p* value, 0.01). Normal distribution was analyzed using the Kolmogorov-Smirnov test. Pearson's correlation analysis (two-tailed) was used for the treatment group only. For grouped subitems of the UPDRS_III_ we corrected for multiple testing with Bonferroni.

## 3. Results

Twenty-six participants with iPD were allocated to the sham group and 30 to the experimental group. One individual of the sham group was excluded from the study on Day 3 due to a change in dopaminergic medication, and one subject in the experimental group withdrew due to a worsening of preexisting back pain; thus, 54 iPD participants completed the trial and were subjected to subsequent analyses. A total of 12 participants reported AEs, although no serious AEs were observed. AEs included muscle soreness (three patients in the experimental group [10.34%]* versus* one in the sham group [4%]), knee joint pain (four [13.79%]* versus* zero [0%]), lower back pain (two [6.9%]* versus* two [8%]), and gastric pain (one [3.45%]* versus* zero [0%]) for the duration of 1 to 2 days. All AEs resolved spontaneously.

Clinical symptoms and disease status at baseline were similar in the sham and experimental groups, with 31 of the 56 participants being classified as fallers (Table 1).

Baseline measures of posturography in the sham group were better than those in the experimental group, but the difference was not significant (*p* = 0.97). Between-group analysis comparing changes (expressed as %) after treatment revealed a significant improvement in postural stability as evaluated by posturography (*p* = 0.03).

**Figure 2:**
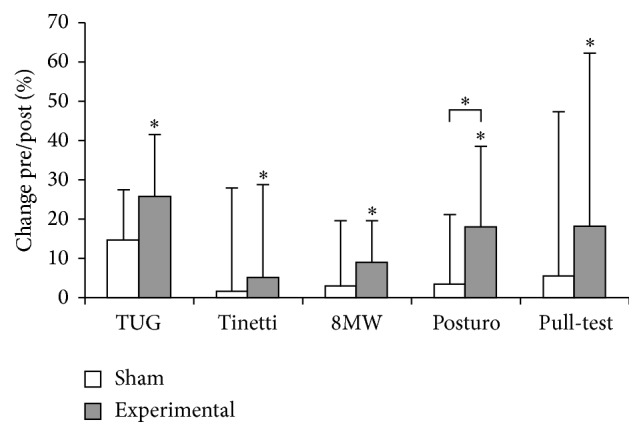
Change (%) of motor scores comparing pre- and posttreatment values. Within-group comparison demonstrated significant improvement in the experimental group (asterisks) but not in the sham group. Between-group analysis comparing change of sham and experimental group was significant for posturography only (asterisk over parenthesis).

Within-group analysis revealed a significant improvement in postural stability, Tinetti sum score, TUG, and 8MW (Figure 2). However, these differences between the sham and experimental groups after treatment were not statistically significant performing the between-group analysis (Table 2).

Within-group analysis revealed improvements in rigidity (*p* < 0.001) and the UPDRS pull-test (*p* < 0.03) in the experimental group but not in the sham group; however, the pull-test results did not pass Bonferroni correction (Table 3). Bradykinesia improved in both groups, but between-group analysis revealed no significant difference in the UPDRS subitems.

Correlation analysis of changes in motor symptoms after treatment and clinical characteristics (i.e., age, sex, duration of disease, dosage of levodopa, and falls) revealed a significant correlation between the Hoehn and Yahr severity stage and an improved Tinetti sum score, the 8MW, and the TUG in the experimental group (Table 4).

The participants' impression of clinical improvement differed between the experimental and sham groups (38%* versus* 20%), whereas 8% of those in the experimental group and 16% in the sham group reported worse symptoms (not assessed in three participants).

## 4. Discussion

The present study provided evidence that SRT can improve postural stability in patients with idiopathic, sporadic PD. Although between-group analyses did not show a significant improvement in bradykinesia, gait, and rigidity-related items, significant differences were observed after within-group comparison. We consider the effect on postural stability to be the most important result. Within-group analysis revealed improved posturography scores, as did between-group analysis of percentage changes after treatment. Additional postural stability-related scores such as the pull-test and 8MW showed improvement only upon within-group analysis, suggesting that the putative sham treatment probably had an effect in itself or that the placebo effect (which is notably strong in PD patients) is even stronger when using a device that exerts a novel bodily sensation. Outcome measures for posturography in the sham group were better than in the treatment groups at baseline but were not significant. We cannot rule out that this influenced our results; however, the improvement in the treatment group was relatively high (17.56%), whereas the changes in the sham group were almost zero.

The study design was based on a standardized mechanical diagnostic technique (dynamic posturography) but also included operationalized timed measurements (TUG and 8MW) and motor function assessments based on clinical rating scales. It is noteworthy that within-group analysis showed that SRT consistently improved gait and postural stability-related scores (e.g., the pull-test, the TUG, the 8MW, the Tinetti gait subscore, and posturography). The improvement in the Tinetti sum score, 8MW, and TUG was positively correlated with Hoehn and Yahr stage (a higher Hoehn and Yahr score was associated with an improved Tinetti sum score, improved walking performance in the 8MW, and improved TUG). Furthermore, the improvement in the Tinetti score correlated with disease duration, implying that severely affected, advanced PD patients also benefit from SRT.

The placebo response in PD studies has been estimated to account for as much as 50% of the total UPDRS_III_ score improvement or two points on at least two UPDRS_III_ items compared to baseline [23]. Correspondingly, in the sham-treated group, we observed a significant improvement in the UPDRS sum score (15.45%) (corresponding to 3.9 points) for speech, an increase of 15.7% for facial items (corresponding to 0.4 points), and an increase of 14.50% for bradykinesia (corresponding to 1.92 points).

To date, six WBV studies have been conducted on PD patients [24]. In contrast to the conflicting results observed with nonstochastic (sinusoidal) stimulation, SRT has shown consistent and positive effects [1, 4, 7, 25–27].

The mechanism underlying the beneficial effects of SRT in PD is unclear. It is hypothesized that dysfunctional proprioceptive regulation in PD is associated with abnormal muscle stretch reflexes, but SRT does not appear to improve proprioceptive performance in PD patients [28]. Our recent preliminary fMRI study showed that SRT activated basal ganglia in young healthy individuals [29], suggesting that SRT may indeed modulate the basal ganglia loops implicated in gait and posture. On the other hand, PET-based measurement of regional cerebral blood flow in individuals suffering from PD showed greater flow in the right cerebellum of PD patients than in that of healthy individuals. Thus, PD patients recruit brain structures not affected by the disease, such as the cerebellum, to compensate for basal ganglia dysfunction [30]. SRT may also stimulate cerebellar loops.

## 5. Study Limitations

Placebo effects were observed for the UPDRS sum score and UPDRS bradykinesia item; this is a common finding in other PD studies. Also, we did not follow up patients after the final treatment, so for how long the positive effects last is unclear. In addition, we do not know whether a longer treatment period (over several weeks) would increase the number of AEs. The small sample size means that the results should be considered preliminary. In general, between-group analysis is more relevant than within-group analysis. Only the outcome measures for posturography (focusing on percentage changes) were significant in between-group analysis.

## 6. Conclusions

Taken together, the results presented herein suggest that SRT is a potential novel treatment option for PD patients suffering from postural instability. In particular, it can be applied to individuals with advanced disease who might not be eligible for more demanding forms of physiotherapy and could thus evolve into a valuable addition to the therapeutic repertoire. However, whether this treatment will reduce the frequency of falls in daily life or for how long the effects last after the treatment has ended remains unclear.

## Figures and Tables

**Table 1:** Demographics and clinical scores of patients at baseline.

	Sham (*n* = 26)	Experimental (*n* = 30)
Male/female	17/9	19/11
Age (y)	67,92 ± 8,78	66,10 ± 8,28
Disease duration (y)	6,96 ± 5,15	7,03 ± 6,48
Hoehn and Yahr stage	2,81 ± 0,80	2,60 ± 0,81
I, *n*	1	3
II, *n*	8	9
III, *n*	12	15
IV, *n*	5	3

UPDRS_III_	25,00 ± 10,46	25,13 ± 13,14
Pull-test	1,50 ± 1,17	1,37 ± 0,89
TUG (s)	11,96 ± 10,47	11,03 ± 9,92
Tinetti score	21,85 ± 7,32	23,17 ± 4,77
8MW (s)	6,67 ± 3,22	6,67 ± 2,58
Fallers/nonfallers, *n* (%)	13 (50,00)	18 (60,00)
Levodopa, *n* (%)	23 (88,46)	24 (80,00)
Levodopa (mg/day)	484,62 ± 311,37	320,83 ± 226,67
Dopamine agonist, *n* (%)	18 (69,23)	25 (83,33)
MAOB inhibitor, *n* (%)	16 (61,53)	16 (50)
Anticholinergic, *n* (%)	1 (3,84)	0 (0)
Nootropic drugs, *n* (%)	4 (15,38)	1 (3,33)

Comparing control and disease group no statistically significant differences were detectable.

**Table 2:** Effect of whole body vibration after treatment (between-group comparison).

	Mean	Difference	*p *value	95% CI
Posturography sham [mm]	263.86 ± 64.26	−30.00	0.14	−47.33, 95.93
Posturography experimental	293.86 ± 144.50			

TUG sham [sec.]	10.46 ± 7.73	+2.15	0.18	−5.38, 1.07
TUG experimental	8.31 ± 3.65			

Tinetti sum score sham	21.92 ± 7.61	+2.32	0.97	−0.49, 5.70
Tinetti sum score experimental	24.24 ± 3.30			

8MW sham [sec.]	6.53 ± 3.03	+0.42	0.43	−1.92, 0.83
8MW experimental	6.11 ± 1.86			

UPDRS_III_ sum score sham	21.44 ± 10.79	+2.58	0.38	−9.03, 3.54
UPDRS_III_ sum score experimental	18.86 ± 12.53			

UPDRS_III_ speech and facial sham	2.36 ± 1.38	+0.19	0.60	−0.91, 0.53
UPDRS_III_ speech and facial experimental	2.17 ± 1.25			

UPDRS_III_ tremor sham	1.24 ± 1.78	−0.31	0.71	−1.03; 1.49
UPDRS_III_ tremor experimental	1.55 ± 2.69			

UPDRS_III_ bradykinesia sham	11.32 ± 5.78	+1.67	0.33	−5.09; 1.76
UPDRS_III_ bradykinesia experimental	9.65 ± 6.65			

UPDRS_III_ rigor sham	2.04 ± 2.52	−0.13	0.86	−1.37; 1,60
UPDRS_III_ rigor experimental	2.17 ± 2.92			

UPDRS_III_ pull-test sham	1.48 ± 1.15	+0.38	0.19	−0.93; 0.19
UPDRS_III_ pull-test experimental	1.10 ± 0.93			

Values are indicated as mean ± SD. CI, confidence interval; “difference” indicates sham-experimental in absolute values; negative prefix indicates clinically worse; positive prefix indicates clinically improved.

**Table 3:** Effect of whole body vibration at baseline and after treatment (within-group comparison).

	Sham (*n* = 25)			Experimental (*n* = 29)		
	Mean	Difference (%)	*p *value	Mean	Difference (%)	*p *value
Posturography pre [mm]	272,02 ± 59,87	−8,16 (−3,00)	0,483	356,53 ± 212,06	−62,7 (−17,57)	**0,005**
Posturography post	263,86 ± 64,26			293,86 ± 144,50		

TUG pre [sec.]	12,21 ± 10,60	−1.74 (−14,33)	0,064	11,13 ± 10,08	−2.82 (−25,33)	0,071
TUG post	10,46 ± 7,73			8,31 ± 3,65		

Tinetti sum score pre	21,64 ± 7,39	+0.28 (+1,29)	0,631	23,10 ± 4,85	+1.14 (+4,93)	**0,030**
Tinetti sum score post	21,92 ± 7,61			24,24 ± 3,30		

8MW pre [sec.]	6,72 ± 3,28	−0.18 (−2,82)	0,592	6,695 ± 2,62	−0.58 (−8,75)	**0,011**
8MW post	6,53 ± 3,03			6,109 ± 1,86		

UPDRS_III_ sum score pre	25,36 ± 10,51	−3.92 (−15,45)	**0,000**	24,79 ± 13,39	−5,93 (−23,92)	**0,000**
UPDRS_III_ sum score post	21,44 ± 10,79			18,86 ± 12,53		

UPDRS_III_ speech facial pre	2,80 ± 1,41	−0.44 (−15,71)	0,024	2,20 ± 1,61	−0.03 (−1,36)	0,912
UPDRS_III_ speech facial post	2,36 ± 1,38			2,17 ± 1,25		

UPDRS_III_ tremor pre	1,48 ± 1,78	−0,24 (−16,21)	0,327	2,24 ± 2,76	−0,69 (−30,80)	**0,006**
UPDRS_III_ tremor post	1,24 ± 1,78			1,55 ± 2,69		

UPDRS_III_ bradykinesia pre	13,24 ± 5,62	−1,92 (−14,50)	**0,008**	12,66 ± 6,26	−3,01 (−23,77)	**0,001**
UPDRS_III_ bradykinesia post	11,32 ± 5,78			9,65 ± 6,65		

UPDRS_III_ rigor pre	2,72 ± 2,86	−0,68 (−25,00)	0,216	3,72 ± 3,94	−1,55 (−41.66)	**0,001**
UPDRS_III_ rigor post	2,04 ± 2,52			2,17 ± 2,92		

UPDRS_III_ pull-test pre	1,56 ± 1,15	−0.08 (−5,12)	0,491	1,34 ± 0,89	−0,24 (−17,91)	0,032
UPDRS_III_ pull-test post	1,48 ± 1,15			1,10 ± 0,93		

Values are indicated as mean ± SD; “pre” indicates baseline scores, “post” indicates scores after the last treatment. Significant *p* values are bold.

Differences are reported as absolute value and percentage.

**Table 4:** Pearson's correlations (significance) of motor symptoms' change after treatment with age, sex, duration of disease, levodopa dosage, and falls frequency in the treatment group.

	Age	Sex	Duration	Levodopa	Hoehn & Yahr
UPDRS_III_ sum score	−0.471 **(0.010)**	−0.027 (0.887)	−0.056 (0.774)	−0.089 (0.648)	0.166 (0.381)
Pull-test	−0.153 (0.429)	0.062 (0.750)	0.179 (0.361)	−0.270 (0.156)	0.233 (0.215)
Tinetti sum score	−0.044 (0.820)	0.054 (0.782)	−0.424 **(0.024)**	−0.068 (0.724)	0.451 **(0.012)**
Posturography	0.142 (0.518)	−0.030 (0.892)	−0.072 (0.750)	0.152 (0.488)	0.160 (0.419)
8MW	−0.309 (0.518)	−0.088 (0.323)	−0.172 (0.381)	−0.166 (0.388)	0.482 **(0.009)**
TUG	0.030 (0.876)	0.147 (0.446)	0.180 (0.361)	0.153 (0.428)	0.405 **(0.027)**

UPDRS: Unified Parkinson's Disease Rating Scale; 8MW: 8-meter walk; TUG: timed up-and-go; values indicate Pearson's correlation. Change represents the difference between pre- and posttreatment values given in percent. Duration: duration of disease; levodopa: total daily dosage.

## Abbreviations

AEs: Adverse events
iPD: Idiopathic Parkinson's disease
MDS: Movement Disorder Society
8MW: 8-meter walk
PD: Parkinson's disease
SAEs: Serious adverse events
SRT: Stochastic resonance therapy
TUG: Timed up-and-go test
UPDRS_III_: Unified Parkinson's Disease Rating Scale, part III
WBV: Whole body vibration.

## References

[1] Haas C. T., Turbanski S., Kessler K., Schmidtbleicher D. (2006). The effects of random whole-body-vibration on motor symptoms in Parkinson's disease. *NeuroRehabilitation*.

[2] Delecluse C., Roelants M., Verschueren S. (2003). Strength increase after whole-body vibration compared with resistance training. *Medicine and Science in Sports and Exercise*.

[3] Merkert J., Butz S., Nieczaj R., Steinhagen-Thiessen E., Eckardt R. (2011). Combined whole body vibration and balance training using Vibrosphere®: improvement of trunk stability, muscle tone, and postural control in stroke patients during early geriatric rehabilitation. *Zeitschrift für Gerontologie und Geriatrie*.

[4] Kaut O., Allert N., Coch C. (2011). Stochastic resonance therapy in Parkinson's disease. *NeuroRehabilitation*.

[5] Kaut O., Jacobi H., Coch C. (2014). A randomized pilot study of stochastic vibration therapy in spinocerebellar ataxia. *Cerebellum*.

[6] Yang F., King G. A., Dillon L., Su X. (2015). Controlled whole-body vibration training reduces risk of falls among community-dwelling older adults. *Journal of Biomechanics*.

[7] Arias P., Chouza M., Vivas J., Cudeiro J. (2009). Effect of whole body vibration in Parkinson's disease: a controlled study. *Movement Disorders*.

[8] Allen N. E., Schwarzel A. K., Canning C. G. (2013). Recurrent falls in Parkinson's disease: a systematic review. *Parkinson's Disease*.

[9] Kerr G. K., Worringham C. J., Cole M. H., Lacherez P. F., Wood J. M., Silburn P. A. (2010). Predictors of future falls in Parkinson disease. *Neurology*.

[10] Shulman L. M., Gruber-Baldini A. L., Anderson K. E. (2008). The evolution of disability in Parkinson disease. *Movement Disorders*.

[11] Boonstra T. A., van der Kooij H., Munneke M., Bloem B. R. (2008). Gait disorders and balance disturbances in Parkinson's disease: clinical update and pathophysiology. *Current Opinion in Neurology*.

[12] Mueller M. C., Jüptner U., Wuellner U. (2009). Parkinson's disease influences the perioperative risk profile in surgery. *Langenbeck's Archives of Surgery*.

[13] Maetzler W., Nieuwhof F., Hasmann S. E., Bloem B. R. (2013). Emerging therapies for gait disability and balance impairment: promises and pitfalls. *Movement Disorders*.

[14] Rodriguez-Oroz M. C., Moro E., Krack P. (2012). Long-term outcomes of surgical therapies for Parkinson's disease. *Movement Disorders*.

[15] Johnson L., James I., Rodrigues J., Stell R., Thickbroom G., Mastaglia F. (2013). Clinical and posturographic correlates of falling in Parkinson's disease. *Movement Disorders*.

[16] Ebersbach G., Gunkel M. (2011). Posturography reflects clinical imbalance in Parkinson's disease. *Movement Disorders*.

[17] Hughes A. J., Daniel S. E., Kilford L., Lees A. J. (1992). Accuracy of clinical diagnosis of idiopathic Parkinson's disease: a clinico-pathological study of 100 cases. *Journal of Neurology, Neurosurgery & Psychiatr*.

[18] Visser J. E., Carpenter M. G., van der Kooij H., Bloem B. R. (2008). The clinical utility of posturography. *Clinical Neurophysiology*.

[19] Fahn S., Elton R. L., UPDRS Program Members, Fahn S., Marsden C. D., Goldstein M., Calne D. B. (1987). Unified Parkinson's disease rating scale. *Recent Developments in Parkinson's Disease*.

[20] Macleod A. D., Counsell C. E. (2010). Timed tests of motor function in Parkinson's disease. *Parkinsonism and Related Disorders*.

[21] Kegelmeyer D. A., Kloos A. D., Thomas K. M., Kostyk S. K. (2007). Reliability and validity of the Tinetti mobility test for individuals with Parkinson disease. *Physical Therapy*.

[22] Lamb S. E., Jørstad-Stein E. C., Hauer K., Becker C. (2005). Development of a common outcome data set for fall injury prevention trials: the Prevention of Falls Network Europe consensus. *Journal of the American Geriatrics Society*.

[23] Goetz C. G., Wuu J., McDermott M. P. (2008). Placebo response in Parkinson's disease: comparisons among 11 trials covering medical and surgical interventions. *Movement Disorders*.

[24] Sharififar S., Coronado R. A., Romero S., Azari H., Thigpen M. (2014). The effects of whole body vibration on mobility and balance in Parkinson disease: a systematic review. *Iranian Journal of Medical Sciences*.

[25] Ebersbach G., Ebersbach A., Edler D. (2010). Comparing exercise in Parkinson's disease—the Berlin LSVT®BIG study. *Movement Disorders*.

[26] Chouza M., Arias P., Viñas S., Cudeiro J. (2011). Acute effects of whole-body vibration at 3, 6, and 9 hz on balance and gait in patients with Parkinson's disease. *Movement Disorders*.

[27] Turbanski S., Haas C. T., Schmidtbleicher D., Friedrich A., Duisberg P. (2005). Effects of random whole-body vibration on postural control in Parkinson's disease. *Research in Sports Medicine*.

[28] Haas C. T., Buhlmann A., Turbanski S., Schmidtbleicher D. (2006). Proprioceptive and sensorimotor performance in Parkinson's disease. *Research in Sports Medicine*.

[29] Schneider C., Kaut O., Fließbach K., Wüllner U. Stochastic whole body vibration induced basal ganglia activation.

[30] Payoux P., Brefel-Courbon C., Ory-Magne F. (2010). Motor activation in multiple system atrophy and Parkinson disease: a PET study. *Neurology*.

